# First Report of *Kalmusia variispora* Causing Bark Necrosis and Branch Dieback of Horse Chestnut (*Aesculus hippocastanum* L.)

**DOI:** 10.3390/pathogens15040445

**Published:** 2026-04-20

**Authors:** Miłosz Tkaczyk, Katarzyna Sikora

**Affiliations:** Diagnosticlab IBL, Department of Forest Protection, Forest Research Institute, Braci Leśnej 3, 05-090 Sękocin Stary, Poland; ibl@ibles.waw.pl

**Keywords:** didymosphaeriaceae, urban tree decline, cambial lesions, ITS rDNA sequencing, Koch’s postulates

## Abstract

Horse chestnut (*Aesculus hippocastanum* L.) is a widely planted ornamental and urban tree valued for its aesthetic and ecological functions. In recent years, declining health of horse chestnut in urban environments has been increasingly reported, often associated with a complex of biotic and abiotic stressors. During a health survey of *A. hippocastanum* trees growing along an urban road corridor in Warsaw, Poland, extensive bark necrosis and branch dieback were observed. The aim of this study was to identify the causal agent of these symptoms using morphological, cultural, molecular (ITS rDNA), and pathogenicity tests under controlled conditions. Fungal isolates were obtained from necrotic tissues and were consistently identified as *Kalmusia variispora* based on ITS sequence analysis (99.0–99.6% similarity to GenBank references) and characteristic morphology. Pathogenicity tests fulfilled Koch’s postulates, reproducing necrotic lesions and cambial damage similar to those observed in the field. To our knowledge, this is the first documented report worldwide of *K. variispora* infecting *A. hippocastanum*. The findings expand the known host range of this opportunistic Didymosphaeriaceae species and highlight its potential role in bark and wood disease complexes of urban trees. Further research is needed to assess its distribution, genetic diversity, and epidemiological significance in urban forest ecosystems.

## 1. Introduction

Horse chestnut (*Aesculus hippocastanum* L.) is one of the most recognisable and highly valued ornamental tree species used in urban greenery across Central and Western Europe. Due to its spectacular flowering, broad crown architecture, and relatively good adaptation to urban habitat conditions, this species has for centuries been an important component of parks, avenues, and representative public spaces. Horse chestnut trees play a significant ecological role by contributing to microclimate regulation, reducing urban surface overheating, enhancing rainwater retention, and improving air quality [1,2]. In many European cities, these trees form historic avenues of considerable landscape and cultural value, and their loss entails not only economic costs but also the degradation of public space.

Since the beginning of the 21st century, a systematic decline in the health of horse chestnut trees in urban environments has been observed. This phenomenon is multifactorial and results from the combined effects of abiotic stresses (drought, soil salinity, traffic-related pollution, and restricted root space) and the pressure of pathogens and pests [3,4]. Physiological weakening of trees promotes colonisation by opportunistic organisms and increases susceptibility to bark and wood diseases, leading to necrosis, canker formation, and branch dieback. Such damage poses a serious threat to the mechanical stability of street trees and to public safety in urban areas [5].

One of the best documented and most destructive threats to horse chestnut is bleeding canker disease, described as a bark disorder characterised by extensive necrosis and the exudation of dark, sticky fluid from bark fissures [6,7]. The disease involves the death of cambial and vascular tissues, resulting in progressive crown decline and, ultimately, tree mortality. Initially, necroses were attributed to oomycetes of the genus *Phytophthora*; however, molecular studies later demonstrated that in many European countries the primary causal agent is the bacterium *Pseudomonas syringae* pv. *aesculi*, responsible for the epidemic observed since the early 2000s [7,8]. In addition to bacterial pathogens, fungi belonging to the class Dothideomycetes, including members of the family Botryosphaeriaceae, play an important role in wood degradation and shoot dieback of horse chestnut. Species of the genus *Diplodia* and related taxa are known for their opportunistic behaviour and their ability to shift from an endophytic to a pathogenic phase under host stress conditions [9,10]. Recent reports have confirmed the presence of *Diplodia* spp. in branch necroses of *A. hippocastanum* growing in urban environments [5].

The spectrum of biotic factors affecting horse chestnut also includes foliar pathogens such as *Erysiphe flexuosa*, the causal agent of powdery mildew, which leads to premature defoliation [11]. Prolonged weakening resulting from leaf infections may further increase tree susceptibility to secondary bark and wood pathogens. In recent years, attention has also been drawn to less-studied representatives of the family Didymosphaeriaceae [12]. The opportunistic nature of these fungi and their ability to colonise weakened tissues suggest that they may play a significant role in the complex of urban tree decline diseases. In the context of intensifying climate change, which promotes water and temperature stress and thereby increases tree susceptibility to infection [13], detailed identification of the etiological agents responsible for bark and wood necroses in urban horse chestnut trees has become crucial from both phytopathological and urban green space management perspectives.

The aim of the present study was to identify the causal agent of bark necroses and branch dieback in horse chestnut (*Aesculus hippocastanum*) growing along a roadside in Warsaw, using ITS rDNA sequence analysis, detailed morphological characterisation of the isolated strains, and verification of their pathogenicity under controlled conditions in accordance with Koch’s postulates.

## 2. Materials and Methods

### 2.1. Sampling and Fungal Isolation

**Figure 1:**
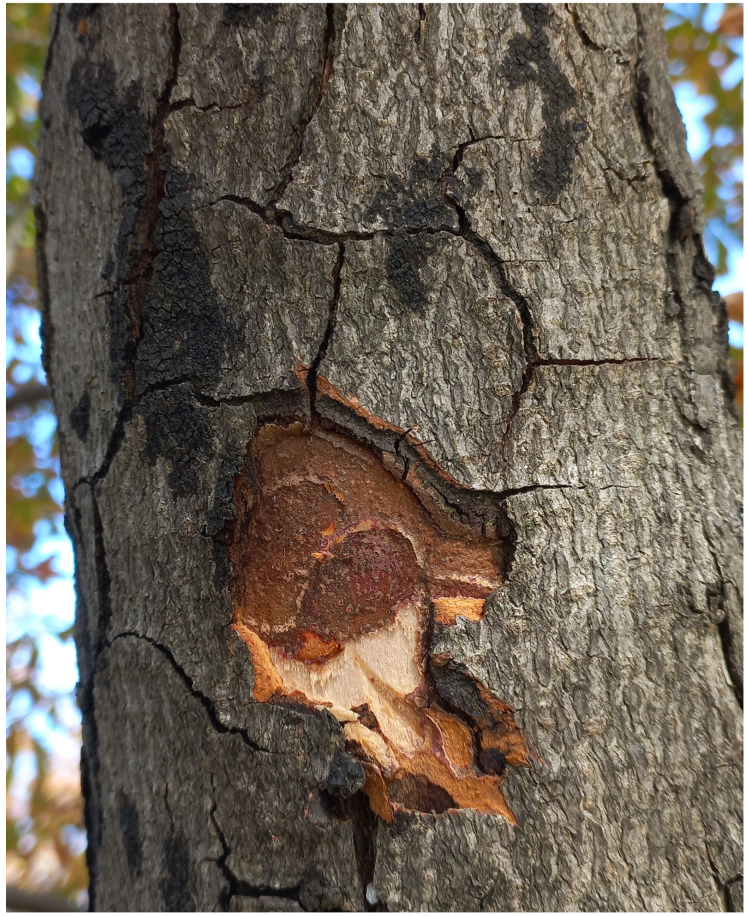
Bark necrosis and branch dieback symptoms observed on *Aesculus hippocastanum* growing along Wołoska Street in Warsaw.

Plant material was collected on 15 October 2025 from horse chestnut trees (*A. hippocastanum*) growing along Wołoska Street in Warsaw, Poland. The examined trees exhibited distinct disease symptoms, manifested as black, irregular lesions on the bark of branches (Figure 1). Branches with necrotic changes gradually declined, as indicated by premature leaf fall and the absence of current-year shoot growth on affected stems. Similar symptoms were observed on nearly all trees (53 individuals) along the surveyed section of the street. After mechanically removing the outer bark layers at the lesion sites, extensive necrosis of the underlying tissues was visible. Four representative trees, spaced approximately 15 m apart, were selected for further analysis to provide a representative assessment of the entire tree row while minimising the potential influence of a localised infection focus. Tissue fragments were excised from the margin between healthy and necrotic tissues. Due to the exploratory nature of this study, a limited number of trees and isolates were selected for detailed analysis. Samples were surface-sterilised in 2% sodium hypochlorite for 1 min, followed by immersion in 70% ethanol for 30 s, and rinsed three times in sterile distilled water. After drying on sterile filter paper, tissue fragments were placed onto Potato Dextrose Agar (PDA; Sigma-Aldrich, Darmstadt, Germany) and incubated at 22–24 °C in the dark. Emerging fungal colonies were purified by single or repeated transfer of actively growing mycelial fragments onto fresh medium until pure cultures were obtained.

### 2.2. Genomic DNA Extraction, PCR, and Sequencing

Genomic DNA was extracted from fresh mycelium grown on malt extract agar (MEA) plates using the Plant DNA Mini Kit (Syngen, Wrocław, Poland), according to the manufacturer’s instructions. The internal transcribed spacer (ITS) region of ribosomal DNA was amplified using the universal primers ITS1F and ITS4, targeting the ITS1–5.8S–ITS2 region [14,15]. Each 25 μL PCR reaction contained 1× PCR buffer (Taq PCR Core Kit, Qiagen, Hilden, Germany), 1.5 mM MgCl_2_, 0.4 mM of each dNTP, 0.2 μM of each primer, 1 U Taq DNA polymerase, and 10–20 ng genomic DNA. The amplification programme consisted of an initial denaturation at 95 °C for 5 min; 35 cycles of 95 °C for 30 s, 56 °C for 30 s, and 72 °C for 50 s; followed by a final extension at 72 °C for 10 min. PCR products were visualised on a 1% agarose gel stained with GelRed^®^ (Biotium, Fremont, CA, USA) and purified using the CleanUp Kit (A&A Biotechnology, Gdańsk, Poland). Sequencing was performed bidirectionally using an Applied Biosystems 3500 Genetic Analyzer (Thermo Fisher Scientific, Waltham, MA, USA). The resulting sequences were compared with reference sequences in the NCBI GenBank database using the BLASTn algorithm (NCBI, 2019) to determine the closest taxonomic matches.

Phylogenetic analysis was performed using the Maximum Likelihood (ML) method implemented in MEGA7 [16]. The Kimura 2-parameter model [17] was applied as the model of nucleotide substitution. The robustness of the inferred tree topology was assessed using 1000 bootstrap replicates [18]. Initial trees for the heuristic search were generated automatically using the Neighbor-Joining [19] and BioNJ [20] algorithms based on pairwise distances estimated with the Maximum Composite Likelihood method [21]. Rate variation among sites was modeled using a discrete Gamma distribution with five categories (+G). All positions containing gaps and missing data were removed (complete deletion), resulting in a final dataset of 441 positions. The analysis included a total of nine nucleotide sequences.

### 2.3. Morphological Characterization

Morphological characterisation was conducted on three representative isolates from a single morphological group. Colonies were cultivated on PDA and MEA (Sigma-Aldrich, Darmstadt, Germany) at 22–24 °C. Radial growth rate, colony colour, surface texture, presence of aerial mycelium, and reverse pigmentation were recorded. To induce the formation of reproductive structures, sterile birch wood fragments were placed on the surface of 7-day-old fungal cultures grown on agar media. Plates were incubated at 24 °C under daylight conditions. The development of fruiting bodies (pycnidia) on the wood fragments was monitored daily for 7 days. Microscopic features, including hyphal structure, morphology of conidiogenous structures, and conidial shape and dimensions, were examined using a ZEISS Axioskop 2 (Carl Zeiss AG, Oberkochen, Germany) and a ZEISS Axiolab microscope equipped with a digital camera, at magnifications ranging from 20× to 400×. Conidial colour, shape, length, and width were measured using Nikon NIS-Elements D software (version 4.50), and minimum, maximum, mean values, and standard deviations were calculated. Colony morphology and pigmentation on different media were assessed according to the colour standards of Rayner [22].

### 2.4. Pathogenicity

The pathogenicity of three selected isolates was evaluated in a greenhouse experiment using three-year-old horse chestnut saplings. Controlled mechanical wounds (5 mm in diameter) were made on the stems with a sterile cork borer under aseptic conditions. The wounds were inoculated with sterile birch wood plugs (5 mm in diameter) previously colonised by the fungus and bearing fruiting bodies. The inoculation sites were sealed with Parafilm (Sigma-Aldrich, Darmstadt, Germany) to prevent contamination and excessive moisture loss. In the control treatment, sterile, non-colonised wood plugs were used as negative controls. The experiment was arranged in a completely randomised design with five plants per (i.e., five replicate trees were used for each isolate) treatment and was repeated once. In total, each isolate was tested on five independent trees per experimental run. Plants were maintained under greenhouse conditions and irrigated by drip watering once or twice per week, depending on water requirements. Two months after inoculation, inoculated plants developed bark symptoms similar to those observed under field conditions, which served as the endpoint of the experiment. Stem fragments including the inoculation sites were excised and subjected to laboratory analysis. After bark removal and surface disinfection, the fungus was re-isolated from symptomatic tissue to confirm its identity and fulfil Koch’s postulates. The assessment of pathogenicity was qualitative and no quantitative measurements of lesion size or statistical analyses were performed.

## 3. Results

### 3.1. Identification of Obtained Isolates

**Figure 2:**
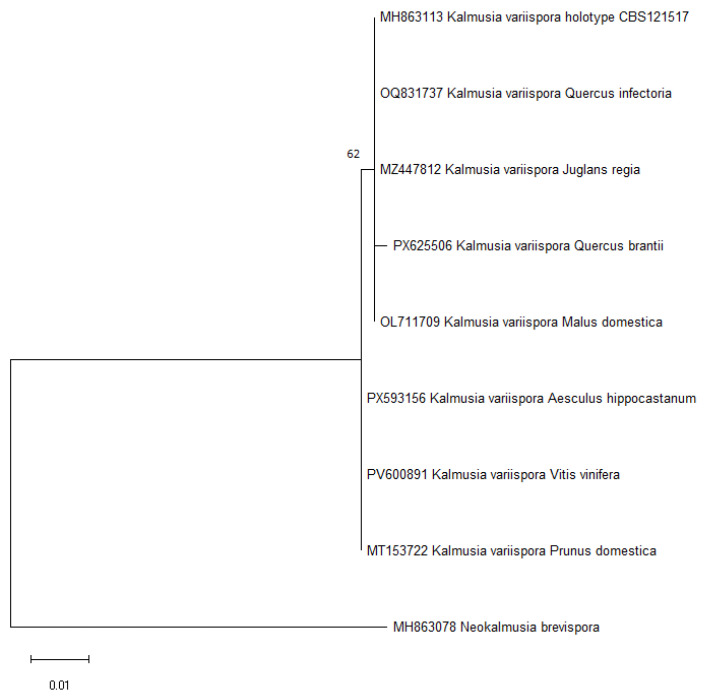
Phylogenetic tree inferred from ITS rDNA sequences using the Maximum Likelihood method based on the Kimura 2-parameter model. The analysis included nine sequences representing *Kalmusia variispora* and one outgroup taxon (*Neokalmusia brevispora*).

High-quality genomic DNA was obtained from all analysed isolates, enabling efficient amplification of the ITS rDNA region. The fungus was consistently isolated from symptomatic tissues collected in the field. PCR amplification produced a single amplicon of approximately 550–600 bp, as confirmed by electrophoresis on a 1% agarose gel. Bidirectional sequencing generated high-quality consensus sequences for each isolate. The obtained sequences were 522 nt in length. BLASTn analysis against the NCBI GenBank database showed the highest similarity (99.0–99.6%) to reference sequences of Kalmusia variispora (Verkley, Göker & Stielow) Ariy. & K.D. Hyde. The best match was sequence PV600891 (isolate from *Vitis vinifera*, Turkey), showing 99.81% identity (522/523 nt; one nucleotide difference) and 100% query coverage. All analysed isolates showed sequence concordance with representatives of this species, without significant intraspecific variation. A representative ITS sequence generated in this study was deposited in GenBank under accession number PX593156. These molecular results confirmed that the isolated pathogen belongs to the species *K. variispora*. Phylogenetic analysis further supported the identification of the isolates (Figure 2). The Maximum Likelihood tree showed that the obtained sequences clustered within the *Kalmusia variispora* clade with high bootstrap support, confirming their taxonomic placement.

### 3.2. Morphological Characterization

**Figure 3:**
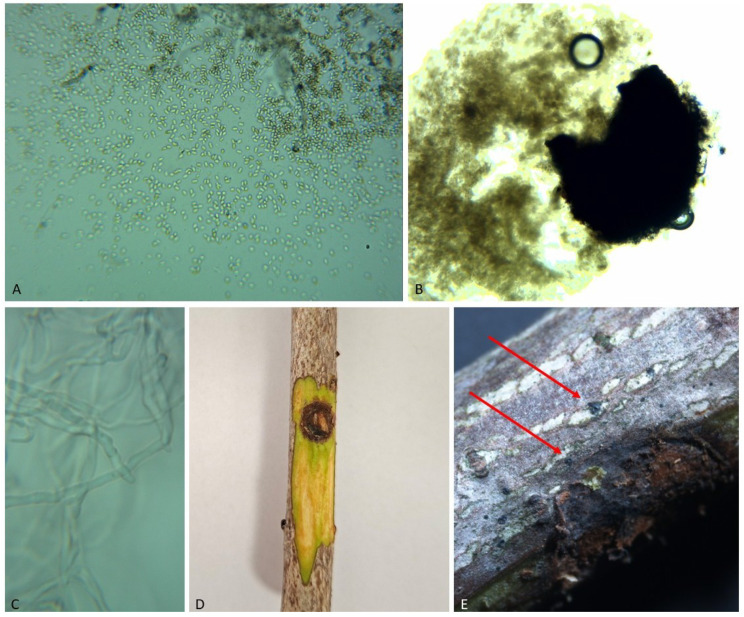
Morphological characteristics and pathogenicity of *Kalmusia variispora*: (**A**) conidia; (**B**) pycnidia formed on sterile birch wood fragments; (**C**) conidiophores (**D**) necrotic lesions on inoculated horse chestnut saplings two months after inoculation; (**E**) red arrows indicate developing pycnidia on the bark surface near the inoculation site.

All isolates assigned to the distinguished morphological group exhibited highly consistent macro- and micromorphological features. On PDA medium, colonies reached a diameter of 52–58 mm after 7 days of incubation at 22–24 °C, indicating a moderately rapid growth rate. Initially, colony surfaces were light grey to olive-grey, gradually darkening to brownish-grey with age. The texture ranged from felty to slightly floccose, with well-developed aerial mycelium in the central region. The reverse side of the colonies was dark brown to nearly black, often with irregular, more intensely pigmented zones. On MEA medium, radial growth was slightly reduced (48–54 mm after 7 days), and colonies displayed a denser structure with limited aerial mycelium. Pigmentation was more uniform, ranging from grey-brown to dark olive. After 14 days of incubation on sterile birch wood fragments, numerous pycnidia developed. These structures were spherical to subspherical, partially immersed in the wood tissue, and measured 180–260 μm in diameter (mean approximately 220 μm). The pycnidial walls were thick and dark brown to black (Figure 3). Conidiophores were short, simple or slightly curved, and hyaline to light brown. Conidia were ellipsoidal to broadly ellipsoidal, initially hyaline and becoming light brown at maturity, smooth-walled, and predominantly unicellular. The biometric parameters obtained were consistent with published descriptions of *Kalmusia variispora*, with minor deviations remaining within the range of intraspecific variability. The combination of macroscopic and microscopic characteristics, together with molecular data, confirmed the identification of the isolates as *K. variispora*. The biometric parameters obtained were consistent with published descriptions of *K. variispora* [23,24], particularly in terms of conidial size, shape, and pycnidial structure, with minor deviations remaining within the range of intraspecific variability. The combination of macroscopic and microscopic characteristics, together with molecular data, confirmed the identification of the isolates as *K. variispora*.

### 3.3. Pathogenicity

Two months after inoculation, distinct disease symptoms developed on the stems of three-year-old horse chestnut saplings at the inoculation sites. Necrotic lesions formed around the wounds, affecting both the bark and underlying tissues (Figure 3). The lesions appeared as dark brown to black, irregularly shaped areas that progressively expanded over time. After removal of the bark at the inoculation sites, necrosis of the cambium and adjacent wood tissues was observed, closely resembling the symptoms recorded in mature trees under field conditions. In contrast, no symptoms or tissue discolouration were observed in the control treatment, where sterile wood fragments were applied. The fungus was consistently re-isolated from symptomatic tissues, and the recovered cultures displayed morphological characteristics identical to those of the original isolates. The identity of the pathogen was confirmed based on colony morphology and reproductive structures, thereby fulfilling Koch’s postulates. The results clearly demonstrate that *K. variispora* is capable of inducing necrotic lesions in the bark and subcortical tissues of horse chestnut. The results demonstrate that *K. variispora* is capable of inducing necrotic lesions under controlled conditions.

## 4. Discussion

The present study clearly demonstrated that bark necrosis and branch dieback of *A. hippocastanum* are associated with the presence of *K. variispora*. It should be noted that the number of analysed isolates was limited and that other potential pathogens associated with similar symptoms were not systematically investigated. Therefore, the observed disease may result from a complex of interacting biotic and abiotic factors. Identification was supported by high ITS rDNA sequence similarity to reference data (99.0–99.6%), as well as by morphological characteristics and pathogenicity tests in which disease symptoms were reproduced and the pathogen was successfully re-isolated. These results confirm the pathogenic potential of the species under controlled conditions; however, they do not fully resolve its role under natural conditions, where multiple biotic and abiotic factors may interact. To date, no published reports have documented infection of *A. hippocastanum* by this species; therefore, this study constitutes the first report of its occurrence in association with symptomatic horse chestnut tissues.

The genus *Kalmusia* belongs to the family Didymosphaeriaceae within the class Dothideomycetes, a taxonomic group whose circumscription has been substantially refined in recent years [12,25]. Members of this family have traditionally been isolated mainly from dead wood or as endophytes of woody plants, suggesting a predominantly saprotrophic lifestyle. However, increasing evidence indicates that the boundary between endophytism and pathogenicity is dynamic, and many taxa may express pathogenic potential under conditions of host physiological stress [9,26]. In this context, *K. variispora* (formerly *Dendrothyrium variisporum*) has gained particular attention. It was originally described from isolates obtained from declining grapevines in Syria and from Erica carnea in Switzerland [23]. Its pathogenicity towards grapevine was first experimentally confirmed in Iran [24], and subsequent reports documented its occurrence in vineyards in California and Washington State (USA) as well as in Greece [27,28]. Notably, ref. [29] demonstrated a significantly higher relative abundance of *K. variispora* in grapevine plants exhibiting symptoms of grapevine trunk diseases (GTDs) compared to asymptomatic specimens, suggesting its potential role as an indicator of phytosanitary status under Greek conditions. Available data further indicate that the host range of this species is broader and not limited to grapevine. *K. variispora* has been reported as the primary causal agent of apple core rot in Greece and Chile [30,31]. It has also been isolated as an endophyte from *Rosa* hybrida and *Pinus eldarica* [24]. In Iran, the species has been associated with oak and sweet chestnut decline, accompanied by symptoms such as defoliation, vascular discolouration, and wood necrosis [32,33]. Moreover, it has been detected in necrotic pomegranate wood, although its pathogenicity in that host has not yet been experimentally confirmed [34].

The symptoms observed in this study—black, irregular bark necroses and cambial damage—partially resemble those described for bleeding canker associated with *Pseudomonas syringae* pv. *aesculi* [7,8]. Earlier reports also linked similar symptoms to infections caused by species of *Phytophthora* [6]. However, differences in symptom progression and the absence of characteristic bacterial exudates suggest a distinct pathogenic mechanism in the case of *K. variispora*. Comparable branch necroses have also been described in association with infections by *Diplodia* spp., including cases reported on horse chestnut in Central Europe [5].

In urban environments, horse chestnut trees often show reduced vitality, which facilitates colonisation by opportunistic organisms. Refs. [2,4] demonstrated that trees growing in highly sealed urban sites experience chronic drought stress and root-zone overheating. These conditions may trigger the transition of *K. variispora* from an endophytic to a pathogenic phase, similar to mechanisms described for other Dothideomycetes [25,26].

The use of the ITS region as a diagnostic marker aligns with standard practice in fungal taxonomy [14,35]. Although ITS is widely recognised as the universal DNA barcode for fungi, further phylogenetic analyses incorporating additional loci (e.g., LSU, tef1-α) could provide more detailed insight into the population variability of the isolates and their potential geographic origin. A crucial component of this study was the experimental confirmation of pathogenicity under controlled conditions. The plant cell wall forms a primary structural barrier against microbial invasion, which successful pathogens must overcome through enzymatic degradation [36]. This process involves the secretion of hydrolytic and oxidative enzymes capable of degrading major cell wall components such as cellulose, hemicelluloses, pectins, and lignin, particularly in woody tissues [37]. The activity of these enzymes is considered a key determinant of pathogenicity and fungal aggressiveness [38]. In *K. variispora*, laccase production—an enzyme involved in lignin degradation—may be qualitatively associated with the intensity of wood discolouration and the extent of necrotic lesions. Makris et al. [39] reported variability among isolates in ligninolytic enzyme activity, with one strain lacking the capacity to degrade lignin. All examined isolates produced enzymes capable of degrading cellulose and pectins, underscoring the fundamental importance of these activities in host tissue colonisation. Additionally, *K. variispora* has been shown to synthesise a broad spectrum of phytotoxic metabolites that may contribute to symptom development [40]. It is noteworthy that numerous reports of new host associations are based solely on isolation from necrotic tissues without verification of pathogenicity. In the present study, fulfilment of Koch’s postulates unequivocally confirmed the ability of *K. variispora* to induce the observed disease symptoms, thereby confirming its pathogenic potential under controlled conditions, although this does not exclude the involvement of additional factors under natural conditions.The limitations of the present study should be acknowledged. The number of analysed isolates and experimental replicates was limited, and the pathogenicity assessment was qualitative in nature. In addition, other potential pathogens associated with similar symptoms were not systematically excluded. Therefore, the observed disease symptoms may reflect a complex etiology involving multiple interacting factors rather than a single causal agent.

In conclusion, this research expands current knowledge of pathogens associated with horse chestnut decline. For the first time worldwide, the ability of *K. variispora* to induce bark and cambial necrosis under controlled conditions has been demonstrated. This finding is significant for both phytopathology and urban tree management. In the context of ongoing climate change and increasing environmental stress, the role of opportunistic members of the Didymosphaeriaceae in the complex of urban tree diseases is likely to intensify. Future studies should include monitoring the pathogen’s occurrence in other European regions, assessing its genetic variability, and evaluating its interactions with other microorganisms colonising horse chestnut tissues.

## Data Availability

The original contributions presented in this study are included in the article. Further inquiries can be directed to the corresponding author.
